# Pornography-Watching Disorder and Its Risk Factors Among Young Adults: Cross-Sectional Survey

**DOI:** 10.2196/49860

**Published:** 2025-01-08

**Authors:** Csaba Erdős, Oguz Kelemen, Dávid Pócs, Edit Paulik, András Papp, Edina Horváth, Arbel Golan, Krisztián Széll

**Affiliations:** 1 Department of Public Health University of Szeged Albert Szent-Györgyi Medical School Szeged Hungary; 2 Department of Behavioral Sciences University of Szeged Albert Szent-Györgyi Medical School Szeged Hungary; 3 Cardiology Center Department of Internal Medicine University of Szeged Szeged Hungary; 4 Family Medicine Department University of Szeged Szeged Hungary; 5 Institute of Education Eötvös Loránd University Budapest Hungary

**Keywords:** pornography, Diagnostic and Statistical Manual of Mental Disorders, Fifth Edition, DSM-5, paraphilia, satisfaction, sexual disorder, sexual education, online survey, young adults, online, risk factor, cross sectional, online survey, pornography-watching disorder, social media, web-based questionnaire, internet use, public health, prevention programs, sexual well-being

## Abstract

**Background:**

The widespread availability of internet-based pornography has led to growing concerns about its impact on mental health, particularly among young adults. Despite increasing recognition of problematic pornography use, standardized diagnostic criteria for pornography addiction are lacking.

**Objective:**

This study aimed to address this gap by applying adapted *DSM-5* (*Diagnostic and Statistical Manual of Mental Disorders* [Fifth Edition]) criteria to evaluate “pornography-watching disorder” (PWD) in a large sample of young adults in Hungary. The primary objective was to assess the prevalence of PWD among young adults and identify key risk factors associated with its development using *DSM-5* criteria adapted for pornography use. It also aimed to advance the understanding of PWD as a potential behavioral addiction.

**Methods:**

A cross-sectional web-based survey was conducted between September and December 2018, targeting young adults aged 18-35 years in Hungary. Participants were recruited through social media and the University of Szeged Albert Szent-Györgyi Medical School’s web page. Of the 9397 respondents, 7187 (76.5%) had previously consumed pornography and were included in the analysis. PWD was measured using 10 statements adapted from the *DSM-5* substance use disorder criteria. Multivariable binary logistic regression was used to identify significant predictors of PWD.

**Results:**

The prevalence of PWD in the sample was 4.4% (n=315). Frequent pornography consumption was a significant risk factor, with weekly users (odds ratio [OR] 0.45, 95% CI 0.33-0.62, *P*<.001), monthly users (OR 0.18, 95% CI 0.11-0.28, *P*<.001), and less than monthly users (OR 0.05, 95% CI 0.03-0.10, *P*<.001) showing significantly lower odds compared with daily users as a reference category. Male sex was associated with a higher risk (OR 0.53, 95% CI 0.39-0.72, *P*<.001), as were early exposure to pornography (OR 0.94, 95% CI 0.90-0.98, *P*=.006), paraphilia (OR 3.95, 95% CI 2.37-6.56, *P*<.001), dissatisfaction with sexual life (OR 0.94, 95% CI 0.90-0.98, *P*=.006), difficulty forming personal relationships (OR 0.93, 95% CI 0.88-0.98, *P*=.005), and strong adherence to religious norms (OR 1.12, 95% CI 1.06-1.19, *P*<.001). Protective factors included adequate sexual education (OR 0.67, 95% CI 0.53-0.87, *P*=.02) and residing in the capital (OR 0.52, 95% CI 0.30-0.91, *P*=.02). The use of an anonymous web-based questionnaire likely reduced the influence of stigma, resulting in more accurate self-reporting of sensitive behaviors.

**Conclusions:**

This study is among the first to apply *DSM-5* criteria to evaluate PWD, providing important insights into its prevalence and associated risk factors in young adults. The findings highlight the need for standardized diagnostic tools for PWD and suggest targeted interventions, particularly for high-risk groups. These results contribute to the ongoing discussion about whether pornography addiction should be recognized as a distinct behavioral disorder.

## Introduction

### Background

The use of pornography has increased significantly in the last few decades, in parallel with the development of the technological means of its transmission, so that access is much easier nowadays than before. Internet pornography shows an increasing trend worldwide, according to Pornhub statistics [1]. To obtain exact data is almost impossible since the number of websites providing erotic content intended for adults is untraceable, and there are no exact data available regarding their use. The massive and ongoing increase in internet use, and internet porn use in particular, has had a profound effect on human sexual behavior over the last decades and related social dynamics [2]. It is estimated that 46%-74% of men and 16%-41% of women in developed countries are active pornography users [3]. Consumption on a weekly basis was found in approximately 50% of young adults under the age of 25, with a general decrease in pornography use with increasing age. According to a study performed in Australia, 84% (8442/10,049) of men from a representative sample population watched porn in their lifetime, while weekly porn consumption was reported in 76% (6131/10,049) in the sample [4]. With the global spread of COVID-19, the use of pornography increased by 4%-24% in several countries in 2020 [5]. Additional features that contribute to the high popularity of internet pornography are the novelty and diversity of the provided contents, which might become a driving force for problematic consumption, as the abundance of sexual imagery can entice further use and lead to tolerance and habituation, well-known in addiction disorders such as alcoholism [6].

Several studies highlight that pornography consumption is often presented as a symptom of sex addiction [7-9]. Individuals with compulsive sexual behavior may misuse the internet in the forms of problematic internet porn use and sex chats as a novel source of sexual gratification, imposing several hazards for themselves and, indirectly, a risk to society [10]. Reid et al [11] found in a field trial on hypersexual disorders that excessive use of pornography was one of the significant behavioral traits of individuals seeking a cure for their disorder, in addition to forced masturbation and frequent casual sex.

Excessive use of internet pornography has emerged as a new type of behavioral addiction, which is in contrast to compulsive sexual behavior disorder being classified as an impulse control disorder rather than an addictive condition in the *ICD-11* (*International Classification of Diseases for Mortality and Morbidity Statistics Eleventh Revision*) [12]. However, the entity pornography addiction is not officially recognized as a disease due to continuous debate among researchers regarding its definition and nature [13]. There is ongoing debate among researchers and clinicians about whether pornography addiction meets the criteria to be considered a distinct mental health disorder. Some researchers contend that compulsive consumption of internet pornography could represent a distinct manifestation of sex addiction or hypersexuality [14], while alternative perspectives advocate for its categorization as a unique subtype of internet addiction [15].

Neither *ICD-11* nor the *DSM-5* (*Diagnostic and Statistical Manual of Mental Disorders,* [Fifth Edition]) provided accepted criteria to identify and define pornography addiction as a type of behavioral addiction so far [16-18].

Reflecting the urgent need for measuring excessive and problematic consumption of pornography characterized by impulsive and compulsive behavior, an alternative definition of self-perceived pornography addiction (SPPA) was suggested [13]. Based on evidence from neurobiological studies demonstrating an overlap between the mechanisms observed in excessive pornography use and those seen in substance addictions, the existing diagnostic criteria for addiction disorders may also be applicable to pornography addiction [19]. Diagnosing addiction to pornography presents challenges due to the subjective nature of defining problematic use, the diversity of individual experiences with pornography, and the lack of clear diagnostic criteria. Without consensus on standardized diagnostic criteria, it is difficult to establish a clear definition of pornography addiction for inclusion in diagnostic manuals.

Numerous procedures are available to measure problematic (compulsive or addictive) use of pornography. Bőthe et al [20] published the Problematic Pornography Consumption Scale (PPCS), which is based on previous addiction models. Nevertheless, research on problematic internet pornography consumption might be limited or lacking due to the absence of formally accepted scales that are compatible with widely accepted diagnostic criteria. Furthermore, 2 scales that aim to assess the addiction characteristics of internet pornography are the Internet Sex Screening Test (ISST) [21] and the Cyber-Pornography Use Inventory (CPUI) [22], both of which work accurately but with limited clinical relevance. The use of compulsive sexual behavior is not suitable for measuring addiction to pornography, as it is an umbrella term that includes many compulsive behaviors and cannot be reduced to specific aspects of pornography [23].

On the other hand, a functional MRI (magnetic resonance imaging)-based study focused on internet pornography addiction revealed similar brain activity patterns in porn addicts as in drug or alcohol addicts. The observation of more robust activation in compulsive sexual behavior than in healthy volunteers was similar to that seen in drug addiction, suggesting neurobiological similarities between the disorders [24].

Defining sexuality-related addictions is challenging because, although sexual addiction and substance dependence share similarities in brain function and behavior, effectively addressing sexuality-related addictions requires a nuanced understanding of the complex interplay among biological, psychological, and social factors that influence human sexuality.

### Objectives of the Study

In the present study, the first aim was to transform the substance use disorder criteria of DSM-5 for measuring porn use based on the above finding. This set of criteria was then used to measure and characterize what we call here “pornography-watching disorder” (PWD) in a young adult population. Such a new measurement method seems to be necessary because the previous methods examined excessive porn use as a behavioral problem, while in the present study, the clinically more relevant criteria of disorder could be assessed. Our second aim was to find relationships between personal features and PWD, which can help future prevention.

## Methods

### Study Design and Recruitment

The cross-sectional exploratory study was conducted using a web-based questionnaire between September and December 2018 in Hungary. A convenience sampling technique was applied. The questionnaire link was made available on the Hungarian-language website of the Albert Szent-Györgyi Medical School. The survey was conducted in Hungarian. Participants were recruited through internet channels, including advertisements on social media platforms such as Facebook (Meta) and Instagram (Meta), as well as through announcements at university lectures. Given the widespread use of social media among internet users, it was chosen as a key platform for participant recruitment. Opt-in sampling in this research was essential to detect and define the problem. Because of the stigmatized nature of the problem, the opt-in sample was best suited to enable the research participants to disclose their problems more easily. While specific measures to confirm residency in Hungary were not used, recruitment was primarily targeted at Hungarian residents through the University of Szeged Albert Szent-Györgyi Medical School’s platforms and Hungarian-language content. Our exploratory research could form the basis for a subsequent representative sample study. The target population was young adults (18-35 years old). We focused on this age group because participants are at a similar stage of life, reducing confounding variables. This age range is relevant because it is when individuals are discovering their sexuality and forming lasting habits. It targets a demographic group that is vulnerable to the effects of pornography because of its accessibility and developmental stage. Narrowing the focus will facilitate subsequent comparative analysis with other age groups, providing insights into developmental trajectories and generational trends.

### Survey Development

The study, titled “The Great Sex Test” (in Hungarian: “A nagy szexteszt”), used a comprehensive questionnaire that addressed various topics, including general sexual health, sexual disorders, sexual satisfaction, contraception, sexually transmitted diseases, pornography addiction, and sexual abuse. This paper specifically focuses on the analysis related to pornography. The questionnaire was developed by a multidisciplinary team, including psychiatrists, sociologists, epidemiologists, medical students, as well as a sexologist and an information technology specialist, whose combined expertise ensured the robustness of the tool.

Pilot testing, which included in-depth interviews, was conducted to ensure the clarity and consistency of the questions. Clear instructions were provided to respondents to facilitate accurate responses. The Cantril scale was frequently used to measure attitudes, and validated variables from previous research were incorporated into the question design, ensuring the questionnaire remained concise without compromising quality.

The final questionnaire was created using Google Forms, a widely accessible platform that adheres to Google’s public data protection policies. The responses were collected in an aggregated, anonymized form and downloaded as a Microsoft Excel spreadsheet.

The questionnaire, which was administered on a voluntary basis, included 218 questions covering all topics, including both male and female sexual disorders. The use of adaptive questioning significantly reduced the number of questions presented to each respondent based on their relevance. Before beginning the survey, participants were required to read and agree to a consent form that detailed the study’s objectives, assured data security, and guaranteed anonymity. The consent form also emphasized the importance of providing accurate information.

An email address was required for questionnaire completion to minimize the risk of multiple submissions. The system handled email addresses separately from the questionnaires. After providing their email, participants were directed to the questionnaire interface through a link. The provided email addresses could not be linked to the completed questionnaires. Although IP address tracking or cookies were not used due to platform limitations, respondents were allowed to review and correct their responses before submission using the back button. Participants could also choose to skip questions by selecting “I don’t know” or “I prefer not to answer.” Irrelevant questions were filtered out through adaptive questioning, ensuring that participants only encountered questions pertinent to them (eg, those who had never viewed pornographic content were not asked related questions).

The number of questions per page was thematically determined, with most pages containing 5-6 questions. The total number of pages varied based on the relevance of the questions to the respondent, with the average questionnaire completion time being 24 minutes. Only fully completed and submitted questionnaires were included in the final analysis. A multistep filtering process was used to exclude questionnaires with unrealistic, frivolous, or inappropriate answers, as well as those completed significantly faster than the average time to maintain the integrity of the results.

### Measurements

#### Dependent Variable: Use of Pornography

An adapted version of DSM-5 criteria for substance use disorder was used in this study, omitting the question on withdrawal. The criteria applied to pornography can be classified as non–substance-related addictions. In these disorders, somatic withdrawal symptoms are not observed, and psychological withdrawal symptoms are difficult to differentiate. Withdrawal is typically a consequence of the sudden stop of some substance. A possible alternative, the criteria for gambling disorder, was considered but abandoned as financial gains and losses are irrelevant here. The criteria of *DSM-5* provide standardized, comprehensive, and clinically valuable guidelines. They are integrated into health care systems and have strong research validity. Furthermore, these criteria have been developed through a rigorous process that involves expert consensus and empirical evidence. It is important to note that using these criteria is not intended to create a new health condition but only to better define an existing problem.

The questionnaire used to identify people with PWD consisted of 10 statements. The first 5 statements reflect the loss of control, and the following 5 questions reflect the negative effects associated with pornography use. The respondents had to decide whether the 10 statements were typical of them (0=false and 1=true) in the past year. The answers were summarized by simple addition, resulting in a 0-10 scale (Cronbach α=0.70), where the cutoff point was set at 3. Accordingly, the group of people with PWD included those who stated that 4 or more statements were typical of them in the past year. The cutoff value was the same as for alcohol use disorder in the *DSM-5* criteria [17]. We did not differentiate between mild, moderate, and vigorous disorders. It is important to emphasize that *DSM-5* does not use the term addiction because of its vague definition and negative connotations. Furthermore, addiction is a broader term used to describe physical dependence and psychological compulsion without specific criteria. By using more precise terminology like “substance use disorder,” the *DSM-5* aims to provide more explicit diagnostic criteria and improve clinical communication. Since the PWD criteria were developed using the *DSM-5* criteria, we have avoided the term addiction.

#### Independent Personal Variables

The questionnaire assessed the demographic characteristics of the participants, including biological sex (0=male and 1=female), age (0=18-24 years and 1=25-35 years), place of residence (0=not in the capital and 1=in the capital), the highest educational attainment of the mother or father (corresponding to International Standard Classification of Education [ISCED] 2011: 0-2, 3 without a general certificate of education, 4 with general certificate of education, 5-8 tertiary education), employment status (working, studying, studying and working, and not studying and not working), parental status (1=have children or 0=not), and relationship status (0=single and 1=in a relationship). We also studied family structure in childhood (1=grew up in a 2-parent family or 0=not) and whether the parents in childhood family are divorced (0=no and 1=yes). A 10-point scale was used to examine the respondent's ideological orientation (between 1=conservative and 10=liberal) and degree of religiosity (by responses to the question, “How much do you adhere to religious norms?”; between 1=not respecting religious norms at all and 10=fully respecting religious norms).

Social relationships and attitudes toward others were captured by a 10-point scale variable that measures the difficulty of establishing personal relationships (“How easily do you make a personal relationship with others?”; from 1=with great difficulty to 10=very easily).

Further questions were aimed at the sexual orientation of the respondents (0=nonheterosexual and 1=heterosexual), the age when they had their first sexual intercourse, the experience in their first sexual intercourse (between 1=very bad and 10=very good), the number of sexual intercourses they had in the last year, the use of sexual performance enhancers (0=not used yet and 1=already used), their satisfaction with their sexual life, body, and genitals (between 1=not satisfied at all and 10=completely satisfied), and how sexually attractive they consider themselves (between 1=not at all and 10=completely). Subjective evaluation areas were measured similarly to the Cantril ladder scale [25].

The questionnaire also covered various sexual disorders. For men, delayed ejaculation, premature (early) ejaculation, hypoactive sexual desire disorder, and erectile disorder, while for women, female sexual interest or arousal disorder, genito-pelvic pain or penetration disorder, and female orgasmic disorder were explored. The answers were summarized into a sexual disorder variable describing the participants being affected by some sexual disorder according to the criteria of *DSM-5* (0=no and 1=yes) [26]. Involvement in paraphilias was measured using the 2 criteria found in *DSM-5*. Criterion “A” explores the qualitative nature of paraphilia, while criterion “B” focuses on the negative consequences. According to *DSM-5*, paraphilic disorders can be diagnosed if both diagnostic criteria are met, and this is also how this disorder was defined in the present study (0=no paraphilia and 1=have paraphilia).

We further measured the frequency of masturbation (1=no masturbation, 2=less than monthly, 3=monthly, 4=weekly, and 5=daily), if the respondents used to talk about their sexual life with others (0=no and 1=yes), and their sexual education, that is, if they received adequate and sufficient sexual information before their first sexual intercourse (0=no and 1=yes), and whether they were ever sexually abused (0=no and 1=yes).

The survey examined the age when the respondents first encountered pornographic content, what kind of experience this first time was (between 1=very bad and 10=very good), and how often they watched porn in the last year (1=never in the last 1 year, 2=less than monthly, 3=monthly, 4=weekly, and 5=daily).

### Statistical Analysis

In addition to descriptive statistics and correlation tests (chi-square test and independent samples *t* test), multivariable binary logistic regression was performed using the Enter method (whereby all variables are added to the model simultaneously) to identify characteristics predicting the risk of PWD. Our model-building strategy was guided by finding the most straightforward, yet most robust model. Since the distribution of those with PWD and those not affected in the sample is very unequal, in order to avoid overfitting and find the simplest model, we built our model based on suggestions by Hosmer et al [27]. The final model includes only those variables that were found to be significant at the *P*<.05 significance level. The final model did not include any variables with interaction effects that were professionally relevant (ie, well-founded, informative, interpretable, significant, and improving model fit). The relative importance of the variables included in the final model was determined using Dominance Analysis [28]. All statistical analyses were conducted with IBM SPSS Statistics (version 28.0) for Windows.

### Ethical Considerations

The study protocol was reviewed and approved by the Regional and Institutional Human Medical Biological Research Ethics Committee of the University of Szeged (ethical approval 161/2017-SZTE). Participation in the study was entirely voluntary, and informed written consent was obtained from all participants before their involvement. The study was conducted in accordance with the principles of the Declaration of Helsinki. All data collected were anonymized to ensure the privacy and confidentiality of the participants.

Informed consent was obtained from all participants before their participation in the study. Participants were provided with detailed information about the purpose of the research, the procedures involved, and the nature of the data being collected. They were informed that their participation was voluntary, and that they could withdraw from the study at any time without any consequences. The consent form also assured participants of the confidentiality of their data, which was anonymized during analysis to protect their privacy. The original informed consent explicitly included provisions for the use of the collected data in subsequent analyses, including secondary research. Therefore, no additional consent was required for the secondary analysis performed in this study, as it was covered by the initial consent obtained from the participants.

The privacy and confidentiality of all participants were rigorously protected throughout the study. All data collected through the web-based survey were anonymized, ensuring that no personally identifiable information was linked to the responses. The survey platform used, Google Forms, provided data in a deidentified format, further ensuring that individual participants could not be identified. The anonymized data were securely stored on the internal servers of the University of Szeged Albert Szent-Györgyi Medical School’s Department of Public Health and Department of Behavioral Sciences with access restricted to authorized research personnel only. In compliance with ethical guidelines, no personal information, such as names or IP addresses, was collected or stored, thereby maintaining the highest level of confidentiality for the study participants.

To encourage participation, tangible incentives such as theater tickets, movie tickets, and spa passes were offered. These prizes were raffled among participants after the data collection was completed. To ensure transparency and fairness in the compensation process, participants were informed about the raffle at the beginning of the survey. Winners were selected randomly and notified through the email addresses they provided.

## Results

A total of 9397 individuals completed the questionnaire. Of these, 7187 had previously consumed pornographic content, and only these participants were included in the present analysis.

A detailed description of the study sample is presented in Tables 1 and 2. The sample consisted of 2398 men and 4789 women. Most of the respondents lived in rural areas and had a relationship. More than one-tenth of the participants (782/7187, 10.9%) have used sexual performance enhancers, while some form of paraphilia was detected in 1.5% (108/7187). Most of them felt that they received adequate sexual information before their first sexual intercourse (5030/7187, 70%). Almost a quarter of them (1631/7187, 22.7%) had not watched porn in the past year, while a quarter of them (1748/7187, 24.3%) did that less often than monthly, less than one-fifth of them (1234/7187, 17.2%), monthly, three-tenths of them (2223/7187, 30.9%), weekly. Every 20th respondent (351/7187, 4.9%) watched porn every day (Table 1).

**Table 1:** Distribution of categorical variables in the affected and nonaffected subgroup of the study sample and univariate associations of these variables with pornography-watching disorder.

Variables				Pornography-watching disorder absent (n=6872), n (%)		Pornography-watching disorder present (n=315), n (%)		Total sample (N=7187), n (%)		Chi-square (*df*)		*P* value		Φ^a^
**Sociodemographic background**														
	**Biological sex**								170.6 (1)		<.001		0.15	
		Male	2186 (91.2)		212 (8.8)		2398 (33.4)							
		Female	4686 (97.8)		103 (2.2)		4789 (66.6)							
	**Age range**								0.02 (1)		.90		—^b^	
		18-24 years	5237 (95.6)		241 (4.4)		5478 (76.2)							
		25-35 years	1635 (95.7)		74 (4.3)		1709 (23.2)							
	**Place of residence**								7.9 (1)		.005		0.03	
		Not in the capital	6251 (95.4)		301 (4.6)		6552 (91.2)							
		In the capital	621 (97.8)		14 (2.2)		635 (8.8)							
	**Father’s highest educational attainment**								0.6 (3)		.89		—	
		Primary education (ISCED^c^ 0-2)	690 (96)		29 (4)		719 (10)							
		Lower secondary education (ISCED 3)	2836 (95.7)		127 (4.3)		2963 (41.2)							
		Upper secondary education (ISCED 4)	1927 (95.3)		94 (4.7)		2021 (28.1)							
		Tertiary education (ISCED 5-8)	1419 (95.6)		65 (4.4)		1484 (20.6)							
	**Mother’s highest educational attainment**								3.2 (3)		.37		—	
		Primary education (ISCED 0-2)	799 (96.4)		30 (3.6)		829 (11.5)							
		Lower secondary education (ISCED 3)	1727 (95.8)		75 (4.2)		1802 (25.1)							
		Upper secondary education (ISCED 4)	2483 (95.1)		128 (4.9)		2611 (36.3)							
		Tertiary education (ISCED 5-8)	1863 (95.8)		82 (4.2)		1945 (27.1)							
	**Employment status**								1.3 (3)		.72		—	
		Working	2638 (95.8)		116 (4.2)		2754 (38.3)							
		Studying	2392 (95.8)		106 (4.2)		2498 (34.8)							
		Studying and working	1554 (95.1)		80 (4.9)		1634 (22.7)							
		Not studying and not working	288 (95.7)		13 (4.3)		301 (4.2)							
	**Family structure in childhood**								0.01 (1)		.96		—	
		Single-parent or other family	1886 (95.6)		86 (4.4)		1972 (27.4)							
		2-parent family	4986 (95.6)		229 (4.4)		5215 (72.6)							
	**Parental divorce in childhood**								0.1 (1)		.82		—	
		Parents are not divorced	2834 (95.5)		132 (4.5)		2966 (41.3)							
		Parents are divorced	4038 (95.7)		183 (4.3)		4221 (58.7)							
	**Parental status**								0.4 (1)		.54		—	
		Have no children	1159 (95.9)		49 (4.1)		1208 (16.8)							
		Have at least one child	5713 (95.6)		266 (4.4)		5979 (83.2)							
	**Relationship status**								21.3 (1)		<.001		0.05	
		Single	1562 (93.6)		107 (6.4)		1669 (23.2)							
		In a relationship	5310 (96.2)		208 (3.8)		5518 (76.8)							
**Sexuality**														
	**Sexual orientation**								2.4 (1)		.12		—	
		Nonheterosexual	551 (94.4)		33 (5.6)		584 (8.1)							
		Heterosexual	6321 (95.7)		282 (4.3)		6603 (91.9)							
	**Use of sexual performance enhancer**								8.5 (1)		.004		0.03	
		Not used yet	6140 (95.9)		265 (4.1)		6405 (89.1)							
		Already used	732 (93.6)		50 (6.4)		782 (10.9)							
	**Sexual disorder**								2.0 (1)		.16		—	
		No	5564 (95.8)		245 (4.2)		5809 (80.8)							
		Yes	1308 (94.9)		70 (5.1)		1378 (19.2)							
	**Paraphilia**								111.2 (1)		<.001		0.12	
		No	6791 (95.9)		288 (4.1)		7079 (98.5)							
		Yes	81 (75)		27 (25)		108 (1.5)							
	**Conversation about the sex life**								2.3 (1)		.13		—	
		No	613 (94.5)		36 (5.5)		649 (9)							
		Yes	6259 (95.7)		279 (4.3)		6538 (91)							
	**Adequate and sufficient sexual education**								7.3 (1)		.007		0.03	
		No	2041 (94.6)		116 (5.4)		2157 (30)							
		Yes	4831 (96)		199 (4)		5030 (70)							
	**Sexual abuse**								0.7 (1)		.39		—	
		No	6135 (95.5)		286 (4.5)		6,421 (89.3)							
		Yes	737 (96.2)		29 (3.8)		766 (10.7)							
**Pornography**														
	**Frequency of pornography watching**								438.9 (4)		<.001		0.25	
		Never in the last 1 year	1626 (99.7)		5 (0.3)		1631 (22.7)							
		Less than monthly	1734 (99.2)		14 (0.8)		1748 (24.3)							
		Monthly	1199 (97.2)		35 (2.8)		1234 (17.2)							
		Weekly	2035 (91.5)		188 (8.5)		2223 (30.9)							
		Daily	278 (79.2)		73 (20.8)		351 (4.9)							

^a^Φ: effect size (phi coefficient or Cramer *V*).

^b^Not applicable.

^c^ISCED: International Standard Classification of Education.

**Table 2:** Means of scales in the affected and non-affected subgroup of the study sample, and independent-samples t tests by variables in the study sample.

Variables		Pornography-watching disorder absent (n=6872), mean (SD)	Pornography-watching disorder present (n=315), mean (SD)	*t* test (*df*)	*P* value	Cohen *d*
**Sociodemographic background**						
	Ideological orientation^a^	6.04 (2.08)	5.79 (2.23)	2.09 (7185)	.04	0.12
	Religiosity^b^	1.94 (1.91)	2.21 (2.20)	–2.11 (7185)	.04	0.14
**Social relationships**						
	Building personal relationships^c^	7.19 (2.11)	6.71 (2.37)	3.55 (7185)	<.001	0.23
**Sexuality**						
	Age at first sexual intercourse	16.43 (1.91)	16.74 (2.18)	–2.54 (7185)	.01	0.17
	Experience of first sexual intercourse^d^	6.22 (2.67)	6.61 (2.72)	–2.55 (7185)	.01	0.15
	Number of sexual partnerships in the last year	1.87 (3.03)	2.07 (2.89)	–1.15 (7185)	.26	—^e^
	Satisfaction with sex life^f^	6.75 (2.76)	5.58 (2.95)	6.89 (7185)	<.001	0.42
	Satisfaction with genitals^f^	7.48 (2.36)	6.93 (2.49)	4.07 (7185)	<.001	0.23
	Being sexually attractive^f^	6.54 (2.02)	6.25 (2.16)	2.32 (7185)	.02	0.14
**Pornography**						
	Age at first encounter with pornographic content	13.98 (3.02)	12.56 (2.90)	8.22 (7185)	<.001	0.47
	Experience of the first encounter with pornographic content^d^	6.20 (2.27)	7.09 (2.29)	–6.77 (7185)	<.001	0.39

^a^1=conservative; 10=liberal.

^b^1=not respecting religious norms at all; 10=fully respecting religious norms.

^c^1=with great difficulty; 10=very easily.

^d^1=very bad; 10=very good.

^e^Not applicable.

^f^1=not satisfied at all; 10=completely satisfied.

Responses to questions on a 10-point scale presented in Table 2 were not normally distributed. Nevertheless, after careful consideration, the means with SD and results of the 2-sample *t* tests are presented. The following were our rationale for this decision. First, a large literature argues that parametric tests can be robust to deviations from the normal distribution, that is, the distribution of the variable is not necessarily and not exclusively the relevant criterion [29-31]. One such criterion may be sample size, that is, for large samples (in our case N=7187), parametric tests are reliable even if the distribution is not perfectly normal. Second, for 5 scales (religiosity, building personal relationships, age at first sexual intercourse, satisfaction with sex life, and being sexually attractive), the variances can be said to differ according to the Levene test. This also means that the results of the Mann-Whitney test may not be robust and should be interpreted cautiously because the test looks at the difference in distributions and not just the medians. Third, the results of parametric tests are easier to interpret and, especially for large samples, parametric tests tend to have greater statistical power than nonparametric tests. The latter means that a nonparametric test is less likely to detect true deviations if they really exist. Fourth, we repeated our analysis using nonparametric tests (Mann-Whitney test) and reached the same conclusions as with the parametric tests presented in the manuscript (independent-sample *t* test). Furthermore, the same conclusions can be drawn from the effect sizes (Cohen *d* and r=rank-biserial correlation coefficient). Most of the respondents considered themselves rather liberal (mean 6.03, SD 2.08, 95% CI 5.98-6.08) and nonreligious, that is, they did not follow religious norms (mean 1.96, SD 1.92, 95% CI 1.91-2.00). They found it relatively easy to establish personal relationships with others (mean 7.17, SD 2.12, 95% CI 7.12-7.22). Their first sexual intercourse usually took place between the age of 16 and 17 years (mean 16.44, SD 1.92, 95% CI 16.40-16.48), which they look back on as a moderately good experience (6.23, SD 2.67, 95% CI 6.17-6.30). Their satisfaction with sexual life was also moderately good (mean 6.70, SD 2.78, 95% CI 6.64-6.76). Satisfaction with the own genital organs showed the highest average value (mean 7.46, SD 2.37, 95% CI 7.40-7.51). The respondents tended to consider themselves sexually attractive but not extraordinarily (mean 6.53, SD 2.03, 95% CI 6.48-6.58). On average, the respondents saw pornographic content first at the age of 14 (mean 13.92, SD 3.03, 95% CI 13.85-13.99), which they had a moderately good experience with (mean 6.24, SD 2.28, 95% CI 6.19-6.29; Table 2).

Among the characteristics listed above, a significant difference was observed between those with PWD and those unaffected by this problem. Concerning all other examined characteristics, no significant differences were detected.

Table 3 displays the number and percentages of individuals who identified the statements related to PWD as true. The regression model’s outcome variable is the presence (1) or absence (0) of PWD. The prevalence of PWD was 4.4% (315/7187). Due to the presence of multicollinearity tested using linear regression (tolerance index<0.20), we excluded 3 variables from the initial set of 32 variables (building partnerships, satisfaction with the own body, and frequency of masturbation) from the model. Thus, a total of 29 independent variables were included in the model. Of the 29 independent variables, 12 examine the sociodemographic background of the respondents, 1 examine their attitudes related to the formation of social relationships, 13 examine their sexual characteristics, and 3 are related to pornography.

**Table 3:** The number and percentage of “true” answers for the pornography-watching disorder-related statements among the respondents (N=7187).

Statement	Answers, n (%)
Pornography is often watched more times or over a longer period than was intended.	698 (9.7)
There is an unsuccessful effort to cut down or control pornography watching.	490 (6.8)
A great deal of time is spent watching pornography.	378 (5.3)
Craving, or a strong desire or urge to watch pornography.	1210 (16.8)
Recurrent pornography watching resulting in a failure to fulfill major role obligations at work, school, or home.	167 (2.3)
Continued pornography watching despite having persistent or recurrent social or interpersonal problems caused or exacerbated by the effects of pornography.	162 (2.3)
Important social, occupational, or recreational activities are given up or reduced because of pornography watching.	62 (0.9)
Recurrent pornography watching in situations in which it is hazardous (eg, in public places, workplaces, and classrooms).	409 (5.7)
Pornography watching is continued despite knowledge of having a persistent or recurrent physical or psychological problem that is likely to have been caused or exacerbated by pornography.	912 (12.7)
Needs to watch an increasing amount or more extreme types of pornography in order to achieve the desired excitement.	185 (2.6)

Based on the likelihood ratio chi-square test, the final binary logistic regression model run to further analyze the relationship between PWD and the background variables examined is significant (for n=7187, *χ*^2^_13_=510.6, *P*<.001); the Hosmer and Lemeshow tests also indicate a suitable model fit (for n=7187, *χ*^2^_8_=7.8, *P*=.46). Based on the Nagelkerke *R*^2^ indicator (0.23), the variable set in the final model explains 22.7% of the variance. Based on the receiver operating characteristics curve analysis, which indicates the probability of true positive classification (sensitivity) as a function of true negative classification (specificity), the area under the curve is 0.84 (cutoff value=0.50; *P*<.001; 95% CI 0.82-0.86), indicating excellent discrimination [27].

In the final model after variable selection, 10 variables were found to be significant (Table 4).

The most powerful factor in the model was the frequency of watching porn. Compared with those who watched porn daily, the odds of having PWD were significantly lower among those who did not watch porn daily (never in the last 1 year, odds ratio [OR] 0.02, 95% CI 0.01-0.06; less than monthly, OR 0.05, 95% CI 0.03-0.10; monthly, OR 0.18, 95% CI 0.11-0.28; and weekly, OR 0.45, 95% 0.33-0.62). A less powerful but still strongly influential factor was biological sex: females were half as likely as males to have the PWD (OR 0.53, 95% CI 0.39-0.72). The other significant variables are of relatively minor importance. The chance of occurrence of PWD is reduced if the participants encountered pornographic content later (OR 0.94, 95% CI 0.90-0.98), were more satisfied with their sexual life (OR 0.94, 95% CI 0.90-0.98), established personal relationships with others more easily (OR 0.93, 95% CI 0.88-0.98), received adequate sexual information before their first sexual intercourse (OR 0.67, 95% CI 0.53-0.87), or lived in the capital (OR 0.52, 95% CI 0.30-0.91). On the other hand, the chance of developing PWD increased if the respondents were affected by some paraphilia (OR 3.95, 95% CI 2.37-6.56) or sexual disorder (OR 2.01, 95% CI 1.45-2.80) and if they followed the norms of their religion as much as possible (OR 1.12, 95% CI 1.06-1.19).

In summary, the predisposing factors for PWD were higher frequency of pornography watching, male sex, occurrence of paraphilia, an early encounter with pornographic content, lower level of satisfaction with sexual life, difficulties in establishing personal relationships, occurrence of a sexual disorder, stronger adherence to religious norms, inappropriate sexual education, and living in the capital.

**Table 4:** Results of binary logistic regression for predicting the likelihood of pornography-watching disorder.

Variables			Odds ratio^a^ (95% CI)		*P* value		Relative importance (%)	
**Frequency of pornography watching (Reference: daily)**								53.94
	Never in the last 1 year	0.02 (0.01-0.06)		<.001				
	Less than monthly	0.05 (0.03-0.10)		<.001				
	Monthly	0.18 (0.11-0.28)		<.001				
	Weekly	0.45 (0.33-0.62)		<.001				
Biological sex (Reference: male)			0.53 (0.39-0.72)		<.001		17.41	
Paraphilia (Reference: absent)			3.95 (2.37-6.56)		<.001		7.48	
Age at first encounter with pornographic content			0.94 (0.90-0.98)		.006		6.84	
Satisfaction with sex life^b^			0.94 (0.90-0.98)		.006		5.59	
Building personal relationships^c^			0.93 (0.88-0.98)		.005		2.13	
Sexual disorder (Reference: absent)			2.01 (1.45-2.80)		<.001		1.90	
Religiosity^d^			1.12 (1.06-1.19)		<.001		1.70	
Adequate and sufficient sexual education (Reference: absent)			0.67 (0.53-0.87)		.002		1.51	
Place of residence (Reference: Capital)			0.52 (0.30-0.91)		.02		1.50	

^a^Values of odds ratio above 1 mean a higher likelihood of inclusion, and values below 1 mean a lower likelihood of inclusion compared with the given reference group. In the table, the variables are listed in descending order of relative importance. Relative importance was determined using dominance analysis.

^b^1=not satisfied at all, 10=completely satisfied.

^c^1=with great difficulty, 10=very easily.

^d^1=not respecting religious norms at all, 10=fully respecting religious norms.

## Discussion

### Principal Findings

This study found that 4.4% of young adults in Hungary exhibit signs of PWD. Key factors contributing to PWD include frequent pornography use, being male, having paraphilic interests, early exposure to pornography, and dissatisfaction with sexual life. Furthermore, individuals who struggle to form personal relationships, experience sexual disorders, strongly adhere to religious norms, received inadequate sexual education, or live in the capital were also more prone to developing PWD.

Our study identified several important risk factors for PWD. The most significant factor was the frequency of pornography consumption, with daily viewers being much more likely to develop PWD than those who watched less often. Sex was also a critical factor, as males were at higher risk. Furthermore, early exposure to pornography and the presence of paraphilia were strongly linked to PWD. In contrast, higher satisfaction with sexual life and greater ease in forming personal relationships were associated with a lower likelihood of developing PWD, indicating these factors might offer some protection against the disorder.

### Comparison With Previous Work

A large-scale survey study of 18,034 participants on gender, sexual orientation, and hypersexuality found a significant association between problematic pornography use and sexual identity because the prevalence was higher among nonheterosexuals than in heterosexuals. The results suggested that GBTQ+ (gay, bisexual, trans, queer, questioning, non-binary or otherwise gender or sexuality-diverse) males have a higher risk of engaging in hypersexual behavior (including high-frequency porn watching) compared with their heterosexual counterparts [32]. The results of our study highlight the role of biological sex, which, in our case, is more relevant than sexual orientation, although the dual association of gender with sexual identity and PWD was not examined.

In connection to religion and ideology, our results suggested a higher prevalence of PWD associated with being religious and having more conservative than liberal views. Both connections were statistically significant. An American study from 2016, on a sample of 713 adult participants, found a higher prevalence of SPPA in religious participants versus nonreligious ones [33], which is in accordance with the current study’s findings.

Regarding relationship status (single or in a relationship), a higher prevalence of PWD was found among singles than among those living in a relationship. Kraus et al [34] examined different characteristics of men interested in finding a treatment for their pornography use within a sample of 1298 male participants and found that more single or unmarried individuals were seeking treatment.

Also related to relationship factors was that people affected with PWD found it, on average, more difficult to form any personal relationship than the control group. Those dissatisfied with sexual education, that is, information obtained before the first sexual intercourse, were also more likely to develop the PWD. These 2 phenomena have apparently not been described in the literature.

Regarding satisfaction with sexual life, the results indicated a significant connection, namely, problematic pornography use was associated with lower levels of satisfaction with sexual life. Results from a study performed on a sample of 832 adults on SPPA, sexual function, and sexual satisfaction indicated that SPPA was associated with sexual dysfunctions and lower sexual satisfaction [35].

### Strengths and Limitations

This study is among the first to apply *DSM-5* criteria to assess PWD in a large sample of young adults, providing a clinically relevant approach to understanding this condition. The large sample size and the inclusion of diverse sociodemographic and psychological variables contribute to the robustness of our findings.

A key strength of the study is the use of a self-report web-based questionnaire with ensured anonymity, which likely reduces the influence of stigma and taboo associated with discussing sensitive topics such as sexuality and pornography. This method also allows for the inclusion of a larger and more diverse sample, enhancing the generalizability of the findings and enabling the application of the modified *DSM-5* criteria to a broader population.

However, the study has several limitations. First, its cross-sectional design limits the ability to draw causal inferences; while associations between certain factors and PWD were identified, cause-and-effect relationships cannot be established.

Second, the study was limited to young adults in Hungary, which may affect the generalizability of the findings to other populations. Cultural differences in attitudes toward pornography and sexual behavior could influence both the prevalence and the risk factors for PWD, suggesting a need for future studies that include participants from diverse geographic locations.

Third, the reliance on self-reported data introduces potential bias, as participants may underreport or overreport their behavior due to social desirability or stigma. Although the anonymity of the survey helps mitigate these issues, future research should consider incorporating more objective measures of pornography consumption and related behaviors.

Fourth, the study did not distinguish between mild, moderate, and vigorous forms of PWD, limiting the granularity of the findings. Future research could benefit from examining the full spectrum of PWD severity to provide a more nuanced understanding of the disorder.

Finally, the use of the Cantril ladder scale for measuring subjective attitudes instead of validated question blocks was chosen to prevent respondent fatigue and maintain the quality of responses. However, this choice, along with differences in data-collection methods compared with other studies, may contribute to variability in the results. Despite these challenges, the study’s methodological approach, particularly the anonymity provided to respondents, remains a valuable tool in exploring sensitive topics like PWD.

### Future Directions

Future research should investigate the longitudinal relationships between early exposure to pornography and the development of PWD to gain a clearer understanding of causality. Furthermore, studies involving more geographically and culturally diverse samples are essential to determine if the risk factors identified in this study are applicable to broader populations. Finally, the development and validation of standardized diagnostic tools for PWD are crucial for advancing our understanding and treatment of this disorder.

### Conclusions

This study characterizes PWD and its prevalence in a large sample population using adapted *DSM-5* criteria based on a web-based survey. The information obtained from this study can be used as a base for further research on pornography consumption and can be used as part of public health prevention programs to advance sexual well-being. In addition, the methods and results of the study can be used to standardize other pornography scales and possibly to supply some evidence required to officially recognize pornography addiction as a disorder. The questionnaire used in this study might enable to development of a web-based interface (website or app) that measures and evaluates the user’s porn consumption and offers them a quiet assistance program in case of need.

## Abbreviations

CPUI: Cyber-Pornography Use Inventory
DSM-5: Diagnostic and Statistical Manual of Mental Disorders (Fifth Edition)
GBTQ+: gay, bisexual, trans, queer, questioning, non-binary or otherwise gender or sexuality-diverse
ICD-11: International Classification of Diseases for Mortality and Morbidity Statistics Eleventh Revision Eleventh Revision
ISCED: International Standard Classification of Education
ISST: Internet Sex Screening Test
MRI: magnetic resonance imaging
OR: odds ratio
PPCS: Problematic Pornography Consumption Scale
PWD: pornography-watching disorder
SPPA: self-perceived pornography addiction

## Data Availability

The datasets generated and/or analyzed during this study are not publicly available due to the fact that this study was conducted using a portion of the database, and ongoing analyses and publications are based on the database, which is in continuous use, but data are available from the corresponding author on reasonable request.
